# 2-Phenyl-4,5-di-*p*-tolyl-1*H*-imidazol-3-ium picrate

**DOI:** 10.1107/S2414314625008776

**Published:** 2025-10-14

**Authors:** Peter Solo, Michael Pillay, M. Arockia doss, Tharsius Raja William Raja

**Affiliations:** a Department of Chemistry, St. Joseph’s College (A), Jakhama, Nagaland, 797001, India; b Department of Environmental Studies, St. Xavier College, Jalukie, Nagaland, India; c Department of Life and Consumer Sciences, College of Agriculture and Environmental Sciences, Florida Campus, University of South Africa, Johannesburg 1709, South Africa; d School of Science and Humanities, St. Joseph University, Emmanuel Educity, Tindivanam-604307, India; e Postgraduate and Research Department of Biotechnology, Bishop Heber College (Autonomous), Tiruchirappalli, Tamil Nadu - 620 017 India; Goethe-Universität Frankfurt, Germany

**Keywords:** crystal structure, imidazolium picrate, hydrogen bonding

## Abstract

The title mol­ecular salt [systematic name: 4,5-bis(4-methylphenyl)-2-phenyl-1*H*-imidazol-3-ium 2,4,6-trinitrobenzen-1-olate], C_23_H_21_N_2_^+^·C_6_H_2_N_3_O_7_^−^, was been synthesized by the slow solvent evaporation method in ethanol and crystallizes in the monoclinic space group *P*2_1_. The structure confirms proton transfer from picric acid to the imidazole ring. The crystal structure is consolidated by multiple N—H⋯O and C—H⋯O hydrogen bonding inter­actions.

## Abstract 2

The title mol­ecular salt, C_23_H_21_N_2_^+^·C_6_H_2_N_3_O_7_^−^, featuring discrete ion pairs, crystallizes from ethanol through slow solvent evaporation. In the crystal, the imidazolium cation and the picrate anion are linked by N—H⋯O hydrogen bonds as a result of proton transfer from picric acid to the pyrimidine-type nitro­gen atom of the imidazole ring.

## Graphical abstract

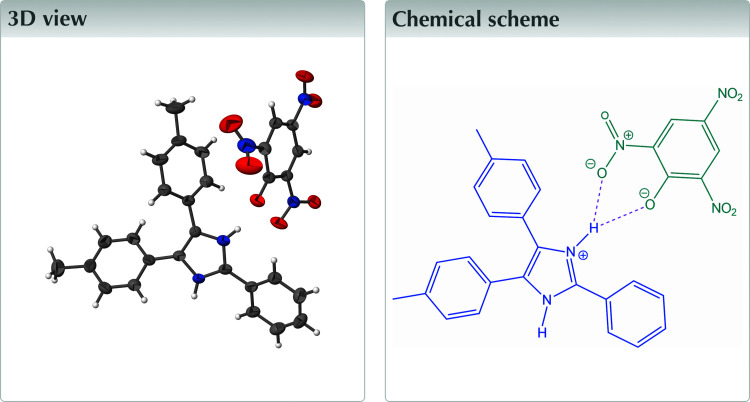

## Structure description

Picric acid has been used for the isolation and purification of basic compounds like alkaloids in the formation of highly stable and crystalline picrate salts (Maldoni, 1991 ▸). Imidazole picrates have been used as energetic materials as the ions can decompose or detonate under shock or heat (Gierczyk *et al.*, 2024 ▸; Mi *et al.*, 2016 ▸). Imidazolium picrate complexes have sharp and high melting points suitable for energetic materials (Smiglak *et al.*, 2012 ▸). A number of imidazolium picrates have already been reported (Anandhi *et al.*, 2011 ▸; Amudha *et al.*, 2017 ▸).

**Figure 1:**
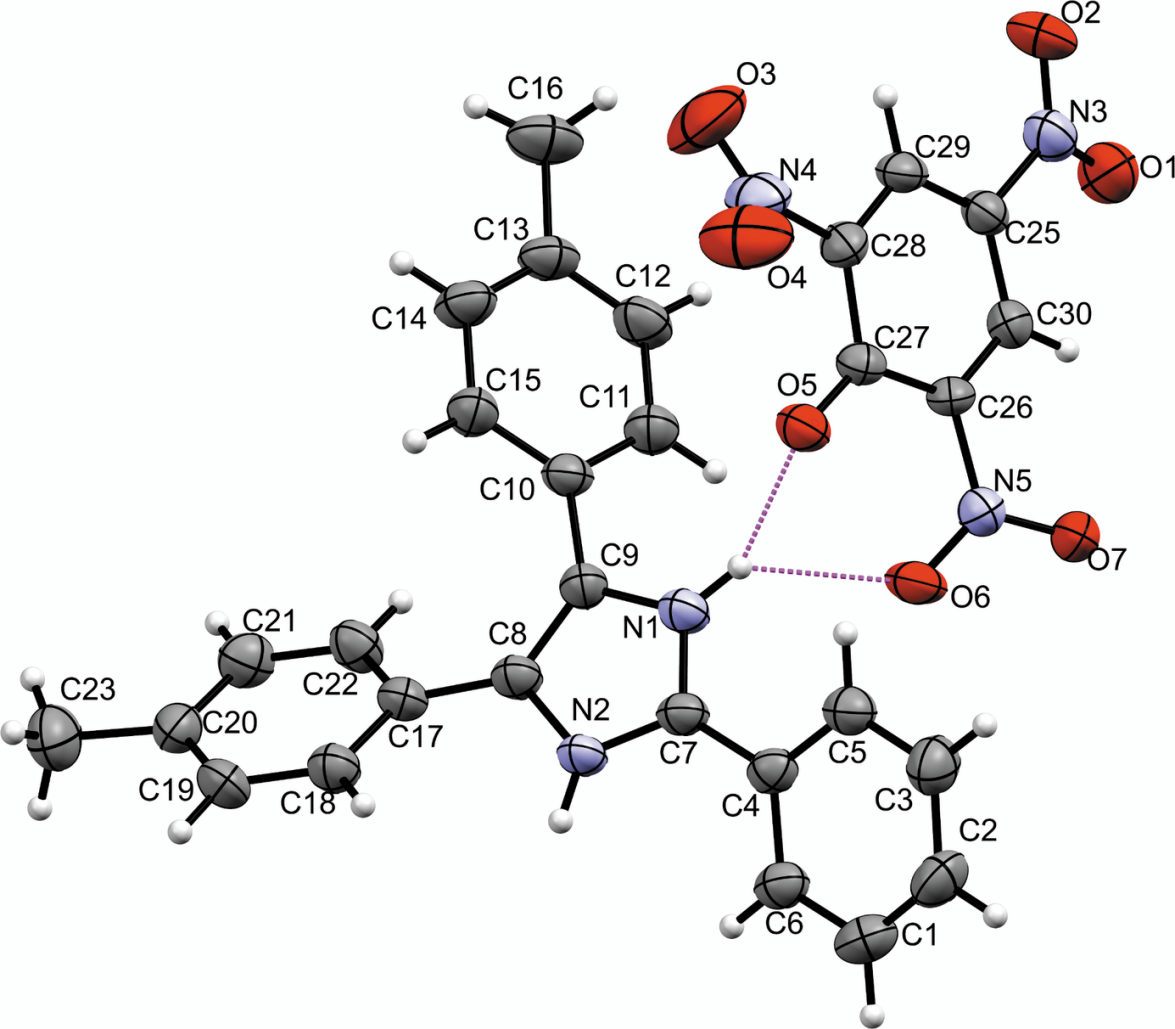
The asymmetric unit of the title compound, with the atomic numbering scheme. Displacement ellipsoids are drawn at the 50% probability level.

**Figure 2:**
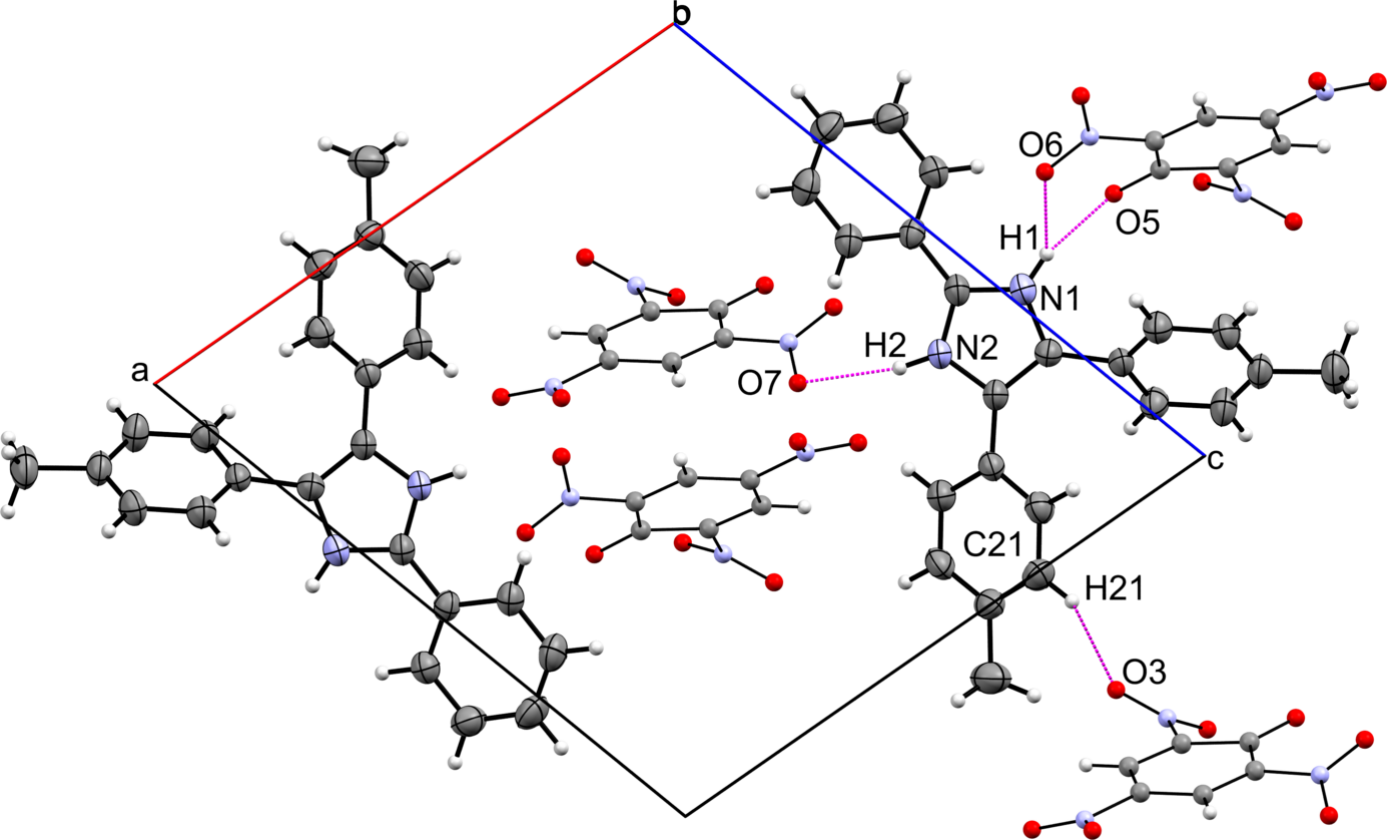
Crystal packing of the title compound with view onto the *ac* plane. The four unique hydrogen-bonding inter­actions in the title compound are shown in magenta.

The structure confirms one proton transfer from picric acid to the pyrimidine-type nitro­gen atom of the imidazole ring. The asymmetric unit of the crystal consist of an imidazolium cation and a picrate anion (Fig. 1 ▸). The dihedral angles between the picrate ring and the other peripheral rings of the cation are 61.9 (2)° (imidazole ring), 78.5 (2)° (C1–C6), 19.9 (2)° (C10–C15) and 68.5 (2)° (C17–C22). This indicates that all the rings are in different planes and therefore we do not observe π–π stacking interactions. Each imidazolium cation is hydrogen bonded to three picrate anions (Fig. 2 ▸). The crystal structure is further consolidated by C—H⋯O hydrogen bonding inter­actions (Table 1 ▸).

## Synthesis and crystallization

**Figure 3:**
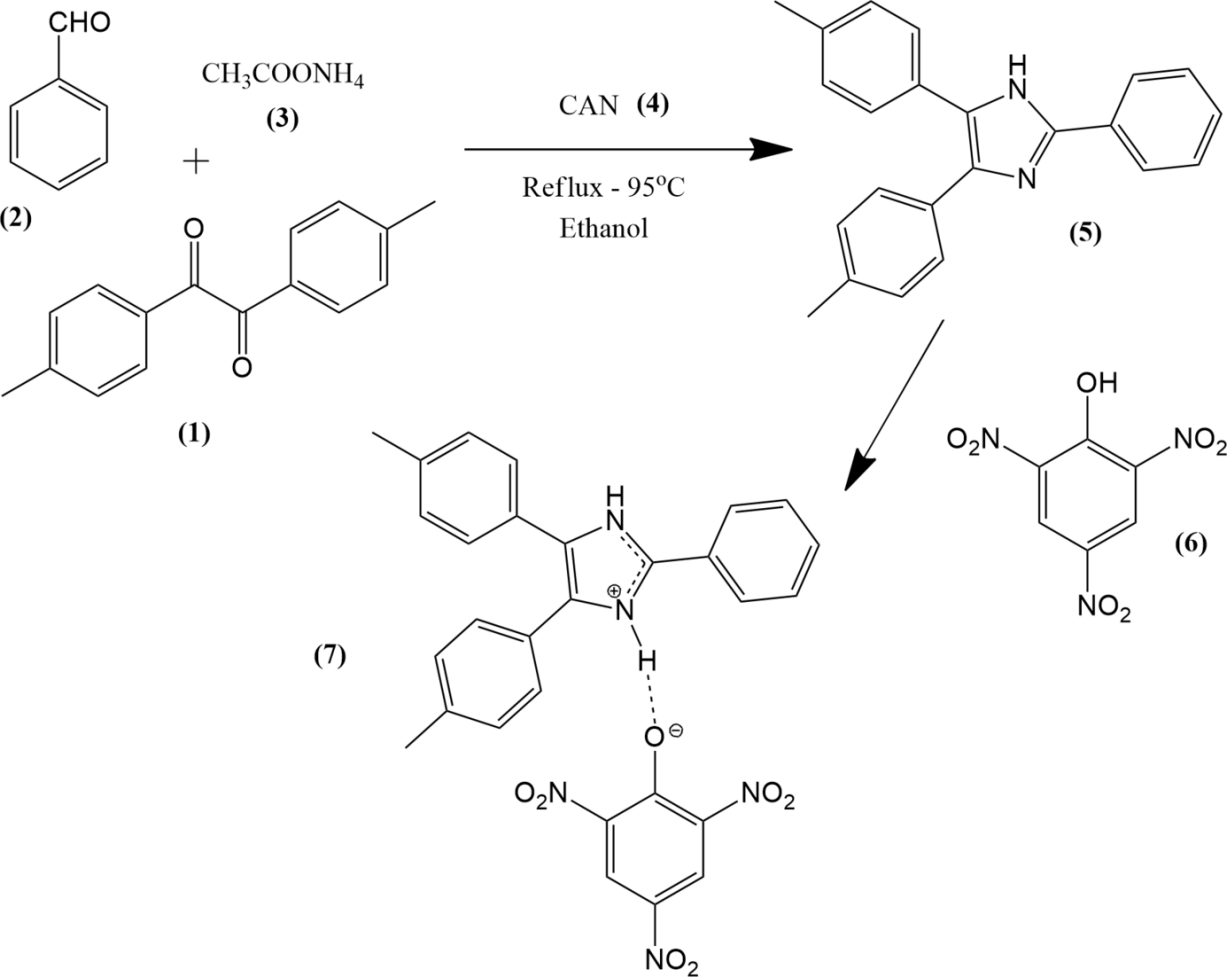
Reaction scheme for the synthesis of the title compound.

The synthesis of the imidazole derivative, 2-phenyl-4,5-di-*p*-tolyl-1*H*-imidazole (**5**), was achieved by a one pot condensation of 4,4-di­methyl­benzil (**1**) (0.953 g, 0.004 moles), benzaldehyde (**2**) (0.424 g, 0.004 moles) and ammonium acetate (**3**) (1.233 g, 0.016 moles) in the presence of ceric ammonium nitrate (CAN, **4**) as catalyst with ethanol as solvent (Fig. 3 ▸). The reflux reaction was carried out at 95°C for about 4 h and the completion of the reaction was monitored using TLC (hexa­ne:ethyl acetate, 1:1). At the end of the reaction the mixture was poured into ice-cold water and the precipitate was collected. The crude precipitate was purified by recrystallization from 90% ethanol solution. Equimolar amounts of 2-phenyl-4,5-di-*p*-tolyl-1*H*-imidazole (**5**) and picric acid (**6**) were mixed in 100% ethanol and heated to 120°C. Imidazolium picrate (**7**) crystals were allowed to grow under slow solvent evaporation.

## Refinement

Details of crystal data, data collection and refinement are given in Table 2 ▸.

## Supplementary Material

Crystal structure: contains datablock(s) I. DOI: 10.1107/S2414314625008776/bt4182sup1.cif

Structure factors: contains datablock(s) I. DOI: 10.1107/S2414314625008776/bt4182Isup3.hkl

Supporting information file. DOI: 10.1107/S2414314625008776/bt4182Isup3.cml

CCDC reference: 2493989

Additional supporting information:  crystallographic information; 3D view; checkCIF report

## Figures and Tables

**Table 1:** Hydrogen-bond geometry (Å, °)

*D*—H⋯*A*	*D*—H	H⋯*A*	*D*⋯*A*	*D*—H⋯*A*
N2—H2⋯O2^i^	0.86	2.64	3.107 (5)	115
N2—H2⋯O7^ii^	0.86	2.18	3.016 (4)	163
N1—H1⋯O5	0.86	1.95	2.727 (5)	149
N1—H1⋯O6	0.86	2.23	2.878 (5)	133
C21—H21⋯O3^iii^	0.93	2.60	3.450 (7)	153

Symmetry codes: (i) 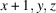; (ii) 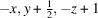; (iii) 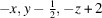.

**Table 2:** Experimental details

Crystal data	
Chemical formula	C_23_H_21_N_2_^+^·C_6_H_2_N_3_O_7_^−^
*M*_r_	553.52
Crystal system, space group	Monoclinic, *P*2_1_
Temperature (K)	296
*a*, *b*, *c* (Å)	12.742 (4), 7.705 (2), 13.799 (4)
β (°)	106.104 (9)
*V* (Å^3^)	1301.6 (7)
*Z*	2
Radiation type	Mo *K*α
μ (mm^−1^)	0.103
Crystal size (mm)	0.54 × 0.37 × 0.21

Data collection	
Diffractometer	Bruker APEXII CCD
Absorption correction	Multi-scan (*SADABS*; Krause *et al.*, 2015 ▸)
*T*_min_, *T*_max_	0.556, 0.746
No. of measured, independent and observed [*I* > 2σ(*I*)] reflections	34163, 6024, 2712
*R*_int_	0.147
(sin θ/λ)_max_ (Å^−1^)	0.664

Refinement	
*R*[*F*^2^ > 2σ(*F*^2^)], *wR*(*F*^2^), *S*	0.049, 0.110, 0.78
No. of reflections	6024
No. of parameters	373
No. of restraints	1
H-atom treatment	H-atom parameters constrained
Δρ_max_, Δρ_min_ (e Å^−3^)	0.19, −0.22
Absolute structure	Flack *x* determined using 899 quotients [(*I*^+^)−(*I*^−^)]/[(*I*^+^)+(*I*^−^)] (Parsons *et al.*, 2013 ▸)
Absolute structure parameter	−0.6 (10)

Computer programs: *APEX2* and *SAINT* (Bruker, 2018 ▸), *OLEX2.solv* (Bourhis *et al.*, 2015 ▸), *SHELXL2025/1* (Sheldrick, 2015 ▸) and *OLEX2* (Dolomanov *et al.*, 2009 ▸).

## full crystallographic data

### 4,5-Bis(4-methylphenyl)-2-phenyl-1H-imidazol-3-ium 2,4,6-trinitrobenzen-1-olate Crystal data

**Table:**

C_23_H_21_N_2_^+^·C_6_H_2_N_3_O_7_^−^	*F*(000) = 576
*M**_r_* = 553.52	*D*_x_ = 1.412 Mg m^−^^3^
Monoclinic, *P*2_1_	Mo *K*α radiation, λ = 0.71073 Å
*a* = 12.742 (4) Å	Cell parameters from 2231 reflections
*b* = 7.705 (2) Å	θ = 3.1–15.6°
*c* = 13.799 (4) Å	µ = 0.10 mm^−^^1^
β = 106.104 (9)°	*T* = 296 K
*V* = 1301.6 (7) Å^3^	Block, clear orangish orange
*Z* = 2	0.54 × 0.37 × 0.21 mm

### 4,5-Bis(4-methylphenyl)-2-phenyl-1H-imidazol-3-ium 2,4,6-trinitrobenzen-1-olate Data collection

**Table:**

Bruker APEXII CCD diffractometer	2712 reflections with *I* > 2σ(*I*)
φ and ω scans	*R*_int_ = 0.147
Absorption correction: multi-scan (SADABS; Krause *et al.*, 2015)	θ_max_ = 28.2°, θ_min_ = 1.5°
*T*_min_ = 0.556, *T*_max_ = 0.746	*h* = −16→16
34163 measured reflections	*k* = −10→10
6024 independent reflections	*l* = −18→18

### 4,5-Bis(4-methylphenyl)-2-phenyl-1H-imidazol-3-ium 2,4,6-trinitrobenzen-1-olate Refinement

**Table:**

Refinement on *F*^2^	H-atom parameters constrained
Least-squares matrix: full	*w* = 1/[σ^2^(*F*_o_^2^) + (0.0002*P*)^2^] where *P* = (*F*_o_^2^ + 2*F*_c_^2^)/3
*R*[*F*^2^ > 2σ(*F*^2^)] = 0.049	(Δ/σ)_max_ < 0.001
*wR*(*F*^2^) = 0.110	Δρ_max_ = 0.19 e Å^−^^3^
*S* = 0.78	Δρ_min_ = −0.22 e Å^−^^3^
6024 reflections	Extinction correction: SHELXL (Sheldrick, 2015), Fc^*^=kFc[1+0.001xFc^2^λ^3^/sin(2θ)]^-1/4^
373 parameters	Extinction coefficient: 0.044 (3)
1 restraint	Absolute structure: Flack *x* determined using 899 quotients [(*I*^+^)-(*I*^-^)]/[(*I*^+^)+(*I*^-^)] (Parsons *et al.*, 2013)
Primary atom site location: iterative	Absolute structure parameter: −0.6 (10)
Hydrogen site location: inferred from neighbouring sites	

### 4,5-Bis(4-methylphenyl)-2-phenyl-1H-imidazol-3-ium 2,4,6-trinitrobenzen-1-olate Special details

**Table:**

Geometry. All esds (except the esd in the dihedral angle between two l.s. planes) are estimated using the full covariance matrix. The cell esds are taken into account individually in the estimation of esds in distances, angles and torsion angles; correlations between esds in cell parameters are only used when they are defined by crystal symmetry. An approximate (isotropic) treatment of cell esds is used for estimating esds involving l.s. planes.
Refinement. The hydrogen atoms were located in a difference map and refined as riding on their parent atom with *U*(H)=1.2*U*_eq_(C,*N*) or with *U*(*H*)=1.5*U*_eq_(C_methyl_). The methyl groups were allowed to rotate but not to tip. Bond lengths were set to C_aromatic_—H=0.93 Å, C_methyl_—H=0.96 Å or N—H=0.86 Å. The reflection 113 with a high error/e.s.d. was omitted. The absolute structure could not be reliably determined.

### 4,5-Bis(4-methylphenyl)-2-phenyl-1H-imidazol-3-ium 2,4,6-trinitrobenzen-1-olate Fractional atomic coordinates and isotropic or equivalent isotropic displacement parameters (Å^2^)

**Table:**

	*x*	*y*	*z*	*U*_iso_*/*U*_eq_	
N2	0.1455 (3)	0.4635 (5)	0.6434 (3)	0.0353 (9)	
H2	0.204991	0.472435	0.625931	0.042*	
C6	0.0959 (4)	0.3741 (6)	0.4234 (4)	0.0415 (13)	
H6	0.162582	0.331685	0.462316	0.050*	
C7	0.0462 (4)	0.4459 (6)	0.5787 (4)	0.0368 (11)	
N1	−0.0236 (3)	0.4355 (5)	0.6348 (3)	0.0386 (10)	
H1	−0.093134	0.423540	0.611141	0.046*	
C4	0.0203 (4)	0.4394 (6)	0.4693 (3)	0.0360 (11)	
C9	0.0310 (3)	0.4467 (6)	0.7360 (4)	0.0363 (11)	
C5	−0.0802 (4)	0.4957 (6)	0.4111 (4)	0.0412 (12)	
H5	−0.132117	0.534895	0.441872	0.049*	
C8	0.1397 (4)	0.4657 (6)	0.7430 (4)	0.0366 (11)	
C19	0.4130 (4)	0.6144 (7)	0.9053 (4)	0.0469 (13)	
H19	0.472630	0.677979	0.898547	0.056*	
C14	−0.0819 (4)	0.5508 (7)	0.9555 (4)	0.0492 (14)	
H14	−0.069251	0.628777	1.008899	0.059*	
C17	0.2351 (3)	0.4888 (6)	0.8283 (4)	0.0364 (11)	
C21	0.3248 (4)	0.4501 (6)	1.0046 (4)	0.0470 (13)	
H21	0.323852	0.403536	1.066524	0.056*	
C3	−0.1037 (4)	0.4940 (7)	0.3068 (4)	0.0518 (14)	
H3	−0.170721	0.534100	0.267071	0.062*	
C20	0.4132 (4)	0.5484 (7)	0.9988 (4)	0.0438 (12)	
C10	−0.0321 (3)	0.4392 (6)	0.8110 (3)	0.0366 (11)	
C13	−0.1652 (4)	0.4350 (7)	0.9426 (4)	0.0444 (13)	
C12	−0.1807 (4)	0.3139 (7)	0.8643 (4)	0.0492 (14)	
H12	−0.235320	0.230534	0.855861	0.059*	
C11	−0.1149 (4)	0.3177 (6)	0.7992 (4)	0.0446 (13)	
H11	−0.126600	0.237484	0.746928	0.054*	
C22	0.2380 (4)	0.4186 (6)	0.9218 (4)	0.0432 (12)	
H22	0.180566	0.349515	0.928332	0.052*	
C18	0.3248 (4)	0.5864 (6)	0.8218 (4)	0.0421 (13)	
H18	0.325491	0.633899	0.760009	0.051*	
C15	−0.0146 (4)	0.5563 (6)	0.8909 (4)	0.0431 (12)	
H15	0.041371	0.637548	0.901175	0.052*	
C1	0.0714 (5)	0.3723 (7)	0.3190 (4)	0.0503 (14)	
H1A	0.122127	0.330259	0.287650	0.060*	
C2	−0.0266 (4)	0.4322 (7)	0.2630 (4)	0.0546 (14)	
H2A	−0.042059	0.431438	0.193043	0.066*	
C16	−0.2424 (4)	0.4338 (8)	1.0085 (4)	0.0632 (16)	
H16A	−0.239347	0.322774	1.040684	0.095*	
H16B	−0.315538	0.455267	0.967645	0.095*	
H16C	−0.221361	0.522742	1.058898	0.095*	
C23	0.5059 (4)	0.5911 (8)	1.0920 (4)	0.0642 (16)	
H23A	0.568917	0.522917	1.091822	0.096*	
H23B	0.483715	0.565050	1.151483	0.096*	
H23C	0.523601	0.712117	1.091616	0.096*	
C27	−0.3190 (3)	0.4287 (6)	0.6040 (3)	0.0333 (10)	
C30	−0.4345 (3)	0.1636 (6)	0.5786 (3)	0.0369 (12)	
H30	−0.450352	0.053799	0.550363	0.044*	
O5	−0.2399 (2)	0.5161 (4)	0.5940 (2)	0.0441 (9)	
O6	−0.1960 (3)	0.2302 (4)	0.5072 (3)	0.0556 (11)	
N5	−0.2878 (3)	0.1788 (5)	0.5019 (3)	0.0398 (10)	
C28	−0.3917 (3)	0.4906 (6)	0.6609 (4)	0.0375 (11)	
O2	−0.6564 (3)	0.2124 (5)	0.6833 (3)	0.0601 (11)	
N3	−0.5892 (3)	0.1346 (6)	0.6499 (3)	0.0466 (11)	
O7	−0.3302 (3)	0.0599 (5)	0.4435 (3)	0.0565 (10)	
C25	−0.4968 (3)	0.2334 (6)	0.6359 (4)	0.0363 (11)	
C26	−0.3495 (3)	0.2567 (6)	0.5636 (4)	0.0330 (11)	
O1	−0.5950 (3)	−0.0189 (5)	0.6298 (3)	0.0656 (11)	
C29	−0.4759 (4)	0.3960 (6)	0.6778 (4)	0.0380 (12)	
H29	−0.518172	0.441016	0.717033	0.046*	
N4	−0.3740 (4)	0.6648 (6)	0.7051 (4)	0.0566 (13)	
O4	−0.3466 (4)	0.7787 (5)	0.6561 (4)	0.0891 (16)	
O3	−0.3909 (4)	0.6874 (6)	0.7847 (4)	0.1012 (17)	

### 4,5-Bis(4-methylphenyl)-2-phenyl-1H-imidazol-3-ium 2,4,6-trinitrobenzen-1-olate Atomic displacement parameters (Å^2^)

**Table:**

	*U*^11^	*U*^22^	*U*^33^	*U*^12^	*U*^13^	*U*^23^
N2	0.029 (2)	0.047 (2)	0.034 (2)	−0.0015 (17)	0.0146 (19)	0.0012 (19)
C6	0.044 (3)	0.042 (3)	0.043 (4)	−0.002 (2)	0.021 (3)	−0.003 (2)
C7	0.035 (3)	0.041 (3)	0.039 (3)	0.000 (2)	0.018 (3)	−0.001 (2)
N1	0.032 (2)	0.045 (2)	0.041 (3)	−0.0023 (18)	0.015 (2)	0.001 (2)
C4	0.039 (3)	0.036 (2)	0.035 (3)	−0.004 (2)	0.016 (2)	0.000 (2)
C9	0.034 (3)	0.043 (3)	0.033 (3)	−0.003 (2)	0.011 (2)	−0.002 (2)
C5	0.039 (3)	0.043 (3)	0.044 (3)	0.002 (2)	0.015 (3)	−0.001 (2)
C8	0.034 (3)	0.043 (3)	0.035 (3)	−0.002 (2)	0.015 (2)	0.002 (2)
C19	0.032 (3)	0.058 (3)	0.053 (4)	−0.007 (2)	0.015 (3)	−0.006 (3)
C14	0.055 (3)	0.054 (3)	0.049 (4)	−0.003 (3)	0.031 (3)	−0.004 (3)
C17	0.031 (2)	0.042 (3)	0.039 (3)	−0.001 (2)	0.014 (2)	−0.002 (2)
C21	0.049 (3)	0.053 (3)	0.039 (3)	0.003 (3)	0.013 (3)	0.008 (3)
C3	0.052 (3)	0.059 (3)	0.040 (3)	0.004 (3)	0.006 (3)	−0.003 (3)
C20	0.041 (3)	0.051 (3)	0.038 (3)	0.004 (2)	0.009 (3)	−0.006 (3)
C10	0.031 (2)	0.047 (3)	0.034 (3)	0.005 (2)	0.013 (2)	0.003 (2)
C13	0.044 (3)	0.055 (3)	0.042 (3)	0.004 (3)	0.024 (3)	0.006 (3)
C12	0.040 (3)	0.059 (3)	0.053 (4)	−0.008 (2)	0.021 (3)	0.005 (3)
C11	0.042 (3)	0.053 (3)	0.042 (3)	−0.004 (2)	0.016 (3)	−0.001 (3)
C22	0.037 (3)	0.049 (3)	0.046 (3)	−0.005 (2)	0.014 (3)	0.005 (3)
C18	0.038 (3)	0.050 (3)	0.040 (3)	−0.003 (2)	0.014 (3)	0.001 (3)
C15	0.042 (3)	0.050 (3)	0.041 (3)	−0.009 (2)	0.017 (3)	−0.005 (3)
C1	0.058 (4)	0.053 (3)	0.047 (4)	0.005 (3)	0.027 (3)	−0.004 (3)
C2	0.067 (4)	0.063 (3)	0.035 (3)	0.001 (3)	0.017 (3)	−0.008 (3)
C16	0.060 (3)	0.077 (4)	0.067 (4)	0.005 (3)	0.040 (3)	0.012 (3)
C23	0.053 (4)	0.078 (4)	0.053 (4)	0.004 (3)	0.001 (3)	−0.006 (3)
C27	0.030 (2)	0.035 (2)	0.036 (3)	0.004 (2)	0.011 (2)	0.002 (2)
C30	0.031 (3)	0.043 (3)	0.035 (3)	−0.001 (2)	0.007 (2)	−0.003 (2)
O5	0.0352 (18)	0.0435 (19)	0.056 (2)	−0.0024 (15)	0.0174 (18)	−0.0037 (16)
O6	0.039 (2)	0.052 (2)	0.087 (3)	−0.0079 (17)	0.035 (2)	−0.022 (2)
N5	0.036 (2)	0.042 (2)	0.043 (3)	−0.0043 (19)	0.013 (2)	−0.007 (2)
C28	0.036 (3)	0.034 (2)	0.044 (3)	0.001 (2)	0.012 (2)	−0.007 (2)
O2	0.040 (2)	0.072 (3)	0.077 (3)	0.0015 (18)	0.031 (2)	0.003 (2)
N3	0.039 (3)	0.057 (3)	0.046 (3)	−0.007 (2)	0.015 (2)	0.000 (2)
O7	0.042 (2)	0.071 (3)	0.060 (3)	−0.0120 (19)	0.0192 (19)	−0.031 (2)
C25	0.028 (2)	0.043 (3)	0.038 (3)	−0.001 (2)	0.010 (2)	0.001 (2)
C26	0.030 (3)	0.036 (3)	0.035 (3)	0.004 (2)	0.013 (2)	−0.005 (2)
O1	0.064 (2)	0.064 (3)	0.076 (3)	−0.026 (2)	0.032 (2)	−0.017 (2)
C29	0.030 (2)	0.051 (3)	0.036 (3)	0.008 (2)	0.013 (2)	0.000 (2)
N4	0.054 (3)	0.047 (3)	0.076 (4)	0.004 (2)	0.032 (3)	−0.013 (3)
O4	0.107 (4)	0.046 (2)	0.139 (5)	0.001 (2)	0.074 (3)	−0.002 (3)
O3	0.140 (4)	0.090 (3)	0.098 (4)	−0.033 (3)	0.074 (4)	−0.051 (3)

### 4,5-Bis(4-methylphenyl)-2-phenyl-1H-imidazol-3-ium 2,4,6-trinitrobenzen-1-olate Geometric parameters (Å, º)

**Table:**

N2—H2	0.8600	C12—H12	0.9300
N2—C7	1.337 (5)	C12—C11	1.389 (6)
N2—C8	1.397 (5)	C11—H11	0.9300
C6—H6	0.9300	C22—H22	0.9300
C6—C4	1.387 (6)	C18—H18	0.9300
C6—C1	1.386 (7)	C15—H15	0.9300
C7—N1	1.333 (5)	C1—H1A	0.9300
C7—C4	1.454 (6)	C1—C2	1.355 (7)
N1—H1	0.8600	C2—H2A	0.9300
N1—C9	1.380 (5)	C16—H16A	0.9600
C4—C5	1.379 (6)	C16—H16B	0.9600
C9—C8	1.369 (6)	C16—H16C	0.9600
C9—C10	1.476 (6)	C23—H23A	0.9600
C5—H5	0.9300	C23—H23B	0.9600
C5—C3	1.387 (7)	C23—H23C	0.9600
C8—C17	1.449 (6)	C27—O5	1.252 (5)
C19—H19	0.9300	C27—C28	1.451 (5)
C19—C20	1.387 (7)	C27—C26	1.448 (6)
C19—C18	1.386 (6)	C30—H30	0.9300
C14—H14	0.9300	C30—C25	1.375 (6)
C14—C13	1.360 (7)	C30—C26	1.363 (6)
C14—C15	1.397 (6)	O6—N5	1.218 (4)
C17—C22	1.389 (6)	N5—O7	1.240 (5)
C17—C18	1.392 (6)	N5—C26	1.440 (5)
C21—H21	0.9300	C28—C29	1.370 (6)
C21—C20	1.378 (6)	C28—N4	1.466 (6)
C21—C22	1.373 (6)	O2—N3	1.234 (5)
C3—H3	0.9300	N3—C25	1.460 (6)
C3—C2	1.374 (7)	N3—O1	1.213 (5)
C20—C23	1.522 (7)	C25—C29	1.375 (6)
C10—C11	1.386 (6)	C29—H29	0.9300
C10—C15	1.395 (6)	N4—O4	1.218 (6)
C13—C12	1.399 (7)	N4—O3	1.188 (6)
C13—C16	1.514 (6)		
			
C7—N2—H2	124.5	C17—C22—H22	119.6
C7—N2—C8	111.1 (3)	C21—C22—C17	120.8 (4)
C8—N2—H2	124.5	C21—C22—H22	119.6
C4—C6—H6	120.2	C19—C18—C17	121.5 (5)
C1—C6—H6	120.2	C19—C18—H18	119.3
C1—C6—C4	119.6 (5)	C17—C18—H18	119.3
N2—C7—C4	126.6 (4)	C14—C15—H15	120.2
N1—C7—N2	106.2 (4)	C10—C15—C14	119.5 (4)
N1—C7—C4	127.2 (4)	C10—C15—H15	120.2
C7—N1—H1	124.7	C6—C1—H1A	120.1
C7—N1—C9	110.7 (4)	C2—C1—C6	119.7 (5)
C9—N1—H1	124.7	C2—C1—H1A	120.1
C6—C4—C7	120.1 (4)	C3—C2—H2A	119.1
C5—C4—C6	119.9 (4)	C1—C2—C3	121.7 (5)
C5—C4—C7	120.1 (4)	C1—C2—H2A	119.1
N1—C9—C10	119.1 (4)	C13—C16—H16A	109.5
C8—C9—N1	107.1 (4)	C13—C16—H16B	109.5
C8—C9—C10	133.8 (5)	C13—C16—H16C	109.5
C4—C5—H5	120.0	H16A—C16—H16B	109.5
C4—C5—C3	120.0 (4)	H16A—C16—H16C	109.5
C3—C5—H5	120.0	H16B—C16—H16C	109.5
N2—C8—C17	122.7 (4)	C20—C23—H23A	109.5
C9—C8—N2	104.9 (4)	C20—C23—H23B	109.5
C9—C8—C17	132.4 (4)	C20—C23—H23C	109.5
C20—C19—H19	119.7	H23A—C23—H23B	109.5
C18—C19—H19	119.7	H23A—C23—H23C	109.5
C18—C19—C20	120.6 (5)	H23B—C23—H23C	109.5
C13—C14—H14	119.0	O5—C27—C28	122.5 (4)
C13—C14—C15	122.1 (5)	O5—C27—C26	126.0 (4)
C15—C14—H14	119.0	C26—C27—C28	111.5 (3)
C22—C17—C8	120.4 (4)	C25—C30—H30	120.3
C22—C17—C18	117.3 (5)	C26—C30—H30	120.3
C18—C17—C8	122.2 (4)	C26—C30—C25	119.5 (4)
C20—C21—H21	119.0	O6—N5—O7	121.1 (4)
C22—C21—H21	119.0	O6—N5—C26	120.1 (4)
C22—C21—C20	122.1 (5)	O7—N5—C26	118.8 (4)
C5—C3—H3	120.5	C27—C28—N4	118.6 (4)
C2—C3—C5	119.0 (5)	C29—C28—C27	124.4 (4)
C2—C3—H3	120.5	C29—C28—N4	116.9 (4)
C19—C20—C23	120.5 (5)	O2—N3—C25	117.7 (4)
C21—C20—C19	117.7 (5)	O1—N3—O2	123.7 (4)
C21—C20—C23	121.7 (5)	O1—N3—C25	118.6 (4)
C11—C10—C9	119.3 (4)	C30—C25—N3	119.2 (4)
C11—C10—C15	118.6 (4)	C29—C25—C30	121.5 (4)
C15—C10—C9	122.0 (4)	C29—C25—N3	119.3 (4)
C14—C13—C12	118.4 (4)	C30—C26—C27	124.2 (4)
C14—C13—C16	122.7 (5)	C30—C26—N5	117.3 (4)
C12—C13—C16	118.9 (5)	N5—C26—C27	118.5 (4)
C13—C12—H12	119.9	C28—C29—C25	118.8 (4)
C11—C12—C13	120.2 (4)	C28—C29—H29	120.6
C11—C12—H12	119.9	C25—C29—H29	120.6
C10—C11—C12	121.0 (5)	O4—N4—C28	117.6 (5)
C10—C11—H11	119.5	O3—N4—C28	118.2 (5)
C12—C11—H11	119.5	O3—N4—O4	124.1 (5)
			
N2—C7—N1—C9	0.0 (5)	C22—C17—C18—C19	−0.8 (6)
N2—C7—C4—C6	29.4 (7)	C22—C21—C20—C19	−0.8 (7)
N2—C7—C4—C5	−151.6 (4)	C22—C21—C20—C23	176.5 (5)
N2—C8—C17—C22	−151.9 (4)	C18—C19—C20—C21	2.2 (7)
N2—C8—C17—C18	30.7 (7)	C18—C19—C20—C23	−175.2 (5)
C6—C4—C5—C3	−2.8 (7)	C18—C17—C22—C21	2.1 (7)
C6—C1—C2—C3	−0.5 (8)	C15—C14—C13—C12	2.5 (8)
C7—N2—C8—C9	0.5 (5)	C15—C14—C13—C16	−176.6 (4)
C7—N2—C8—C17	−178.0 (4)	C15—C10—C11—C12	1.1 (7)
C7—N1—C9—C8	0.3 (5)	C1—C6—C4—C7	−178.4 (4)
C7—N1—C9—C10	179.6 (4)	C1—C6—C4—C5	2.5 (7)
C7—C4—C5—C3	178.2 (4)	C16—C13—C12—C11	176.5 (5)
N1—C7—C4—C6	−150.7 (5)	C27—C28—C29—C25	−2.3 (7)
N1—C7—C4—C5	28.4 (7)	C27—C28—N4—O4	38.3 (7)
N1—C9—C8—N2	−0.5 (5)	C27—C28—N4—O3	−144.3 (5)
N1—C9—C8—C17	177.9 (5)	C30—C25—C29—C28	0.7 (7)
N1—C9—C10—C11	43.5 (6)	O5—C27—C28—C29	−176.7 (5)
N1—C9—C10—C15	−133.3 (5)	O5—C27—C28—N4	2.4 (7)
C4—C6—C1—C2	−0.9 (7)	O5—C27—C26—C30	178.0 (5)
C4—C7—N1—C9	−180.0 (4)	O5—C27—C26—N5	−3.2 (7)
C4—C5—C3—C2	1.3 (7)	O6—N5—C26—C27	20.9 (6)
C9—C8—C17—C22	30.0 (8)	O6—N5—C26—C30	−160.2 (4)
C9—C8—C17—C18	−147.4 (5)	C28—C27—C26—C30	−1.0 (6)
C9—C10—C11—C12	−175.8 (4)	C28—C27—C26—N5	177.8 (4)
C9—C10—C15—C14	175.5 (4)	O2—N3—C25—C30	−165.4 (4)
C5—C3—C2—C1	0.3 (8)	O2—N3—C25—C29	12.7 (6)
C8—N2—C7—N1	−0.3 (5)	N3—C25—C29—C28	−177.4 (4)
C8—N2—C7—C4	179.7 (4)	O7—N5—C26—C27	−158.8 (4)
C8—C9—C10—C11	−137.5 (5)	O7—N5—C26—C30	20.1 (6)
C8—C9—C10—C15	45.8 (8)	C25—C30—C26—C27	−0.3 (7)
C8—C17—C22—C21	−175.4 (4)	C25—C30—C26—N5	−179.2 (4)
C8—C17—C18—C19	176.8 (4)	C26—C27—C28—C29	2.4 (6)
C14—C13—C12—C11	−2.6 (8)	C26—C27—C28—N4	−178.6 (4)
C20—C19—C18—C17	−1.4 (7)	C26—C30—C25—N3	178.7 (4)
C20—C21—C22—C17	−1.4 (7)	C26—C30—C25—C29	0.6 (7)
C10—C9—C8—N2	−179.6 (5)	O1—N3—C25—C30	15.6 (6)
C10—C9—C8—C17	−1.3 (9)	O1—N3—C25—C29	−166.2 (5)
C13—C14—C15—C10	−0.5 (8)	C29—C28—N4—O4	−142.6 (5)
C13—C12—C11—C10	0.8 (7)	C29—C28—N4—O3	34.8 (7)
C11—C10—C15—C14	−1.3 (7)	N4—C28—C29—C25	178.6 (5)

### 4,5-Bis(4-methylphenyl)-2-phenyl-1H-imidazol-3-ium 2,4,6-trinitrobenzen-1-olate Hydrogen-bond geometry (Å, º)

**Table:**

*D*—H···*A*	*D*—H	H···*A*	*D*···*A*	*D*—H···*A*
N2—H2···O2^i^	0.86	2.64	3.107 (5)	115
N2—H2···O7^ii^	0.86	2.18	3.016 (4)	163
N1—H1···O5	0.86	1.95	2.727 (5)	149
N1—H1···O6	0.86	2.23	2.878 (5)	133
C21—H21···O3^iii^	0.93	2.60	3.450 (7)	153

Symmetry codes: (i) *x*+1, *y*, *z*; (ii) −*x*, *y*+1/2, −*z*+1; (iii) −*x*, *y*−1/2, −*z*+2.
